# Supplementary material for: Metagenomic sequencing enables accurate pathogen and antimicrobial susceptibility profiling in complicated UTIs in approximately four hours
Source: Nat Commun. 2025 Dec 3;17:187. doi: 10.1038/s41467-025-66865-8 (PMC12780005; doi:10.1038/s41467-025-66865-8)
Supplement: Supplementary file 1 — Supplementary Information [file 41467_2025_66865_MOESM1_ESM.pdf]

Metagenomic sequencing enables accurate pathogen and antimicrobial susceptibility profiling in complicated UTIs in approximately four hours

Anurag Basavaraj Bellankimath[1#], Sverre Branders[1#], Isabell Kegel[2,3], Jawad Ali[1], Fatemeh Asadi[1], Truls E. Bjerklund Johansen[5,6], Can Imirzalioglu[2,3], Torsten Hain[2,3], Florian Wagenlehner[3,4], and Rafi Ahmad[1,7*]

[1]Department of Biotechnology, University of Inland Norway, Holsetgata 22, 2317, Hamar, Norway.

[2]Institute of Medical Microbiology, Justus Liebig University Giessen, Giessen, Germany

[3]German Center for Infection Research (DZIF), Partner Site Giessen-Marburg-Langen, Giessen, Germany

[4]Clinic for Urology, Pediatric Urology and Andrology, Justus Liebig University Giessen, Giessen, Germany

[5]Institute of Clinical Medicine, University of Oslo, Norway

[6]Institute of Clinical Medicine, University of Aarhus, Denmark

[7]Institute of Clinical Medicine, Faculty of Health Sciences, UiT - The Arctic University of Norway, Hansine Hansens veg 18, 9019, Tromsø, Norway

Email: anurag.bellankimath@inn.no, sverre.branders@inn.no , isabell.kegel@med.uni-giessen.de, jawad.ali@inn.no, t.e.b.johansen@medisin.uio.no, can.imirzalioglu@mikrobio.med.uni-giessen.de , torsten.hain@mikrobio.med.uni-giessen.de , florian.wagenlehner@chiru.med.uni-giessen.de , rafi.ahmad@inn.no[*]

Supplementary Table 1: An overview of all the different methods used in the study along with the details of the method combinations. All extractions with the commercial methods were performed according to the manufacturer's instructions.

Method number	Method combination	Abbreviation in manuscript	Method category	Endonuclease s used	Cell lysis combination	DNA extraction
1	Molysis Complete 5	MC5	Commercial	-	According to the standard protocol	Column based
2	Host Zero Microbial DNA extraction kit	HZ	Commercial	-	According to the standard protocol	Column based
3	Naxtra Blood Total nucleic acid kit	NB	Commercial	-	According to the standard protocol	Magnetic beads
4	HL_SAN + Naxtra Blood Total Nucleic acid kit	H + NB	Non-optimized (In-house)	HL_SAN	Enzymatic lysis	Column based
5	M_SAN + Naxtra Blood Total nucleic acid kit	M + NB	Non-optimized (In-house)	M_SAN	Enzymatic lysis	Column based
6	HL_SAN + Blood and Tissue	H + BT	Non-optimized (In-house)	HL_SAN	Chemical lysis	Magnetic beads
7	M_SAN + Blood and Tissue	M + BT	Non-optimized (In-house)	M_SAN	Chemical lysis	Column based
8	HL_SAN + Enzymatic lysis + Naxtra Blood	H + LZ + NB	Non-optimized (In-house)	HL_SAN	Enzymatic lysis + Chemical lysis	Magnetic beads
9	M_SAN + Enzymatic lysis + Naxtra Blood	M + LZ + NB	Non-optimized (In-house)	M_SAN	Enzymatic lysis + Chemical lysis	Magnetic beads
10	HL_SAN + Bead beating + Naxtra Blood	H + BB + NB	Non-optimized (In-house)	HL_SAN	Mechanical lysis + Chemical lysis	Magnetic beads
11	M_SAN + Bead beating + Naxtra Blood	M + BB + NB	Optimized method (In-house)	M_SAN	Mechanical lysis + Chemical lysis	Magnetic beads

Supplementary Table 2: Bacterial genes used for the qPCR and PCR based assays. Table providing an overview of the genes used for detection of the corresponding uropathogens in qPCR/PCR based assays.

Gene	Target pathogen	Forward	Reverse	Reference
UspA	*Escherichia coli*	CCGATACGCTGCCAATCAGT	ACGCAGACCGTAGGCCAGAT	1
Khe	*Klebsiella pneumoniae*	TGATTGCATTCGCCACTGG	GGTCAACCCAACGATCCTG	
UrePa	*Proteus mirabilis*	GGTGAGATTTGTATTAATGG	ATAATCTGGAAGATGACGAG	
phzA2	*Pseudomonas aeruginosa*	GTTTACCGACAACCTGGAA	GCAATAGCCCTGCGGATAC	
GroES	*Enterococcus faecalis*	GGAATTGTTCTTGCATCCGT	ACAATTAAGTATTCTACGCC	

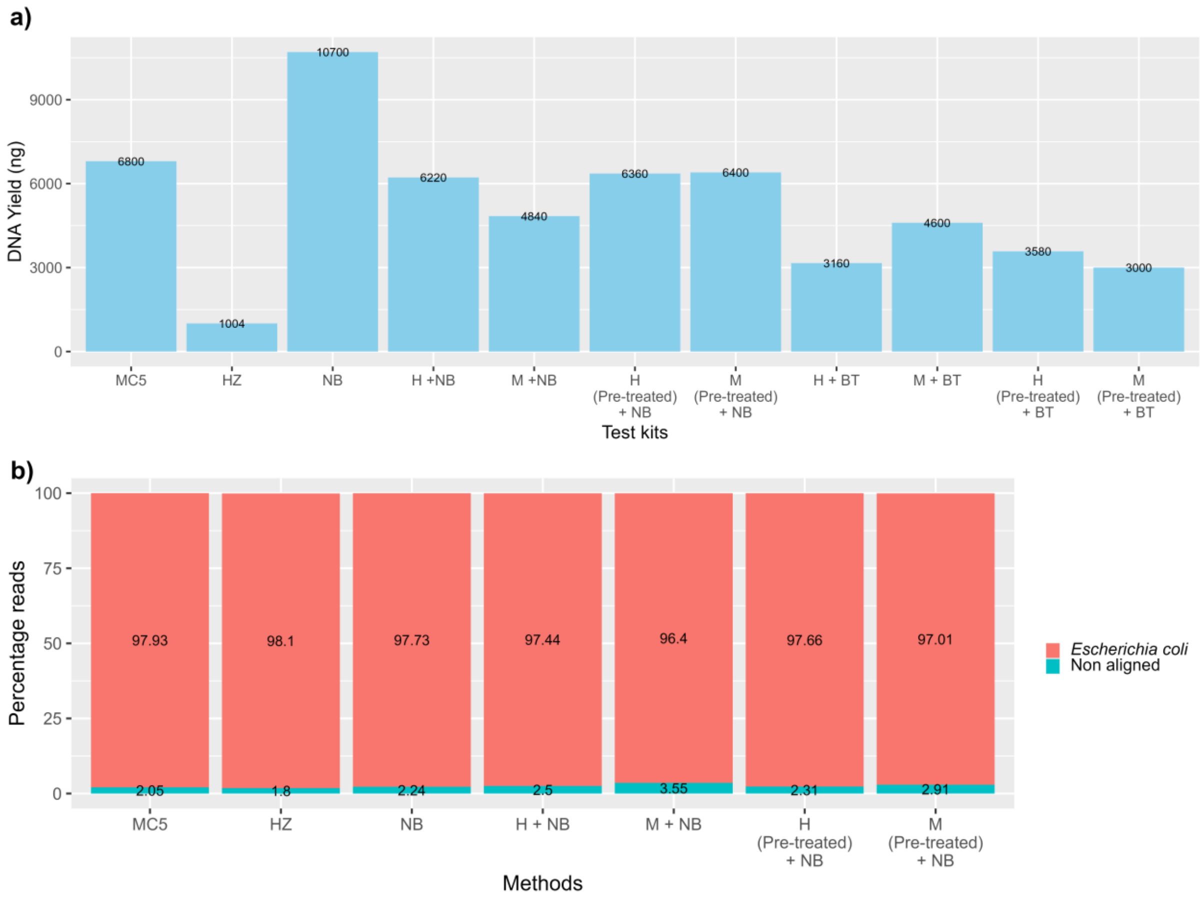

Supplementary Figure 1: DNA yield and sequencing results for spiked samples Subfigure (a) bar graph denoting the DNA yield (ng) obtained from different DNA extraction methods tested on urine samples spiked with a clinical *Escherichia coli* isolate. Sample treatment with EDTA (pre-treatment) had a minor effect on the DNA yield. Subfigure (b) gives an overview of the results of the spike experiment, where the abundance of pathogen (*E. coli*), host, and other prokaryote reads are shown for different DNA extraction methods. Reads were mapped against the NCBI prokaryotic reference genomes database. Reads that did not align to prokaryotic genomes were indicated as 'Nonaligned'. Commercial methods: MC5 (Molysis Complete 5), HZ (Host Zero), and NB (Naxtra Blood Total Nucleic kit). In-house (non-optimized): H (HL_SAN) + NB, M (M_SAN) +NB, H+BT (Blood and Tissue) and M+BT. Source data are provided as a Source Data file.

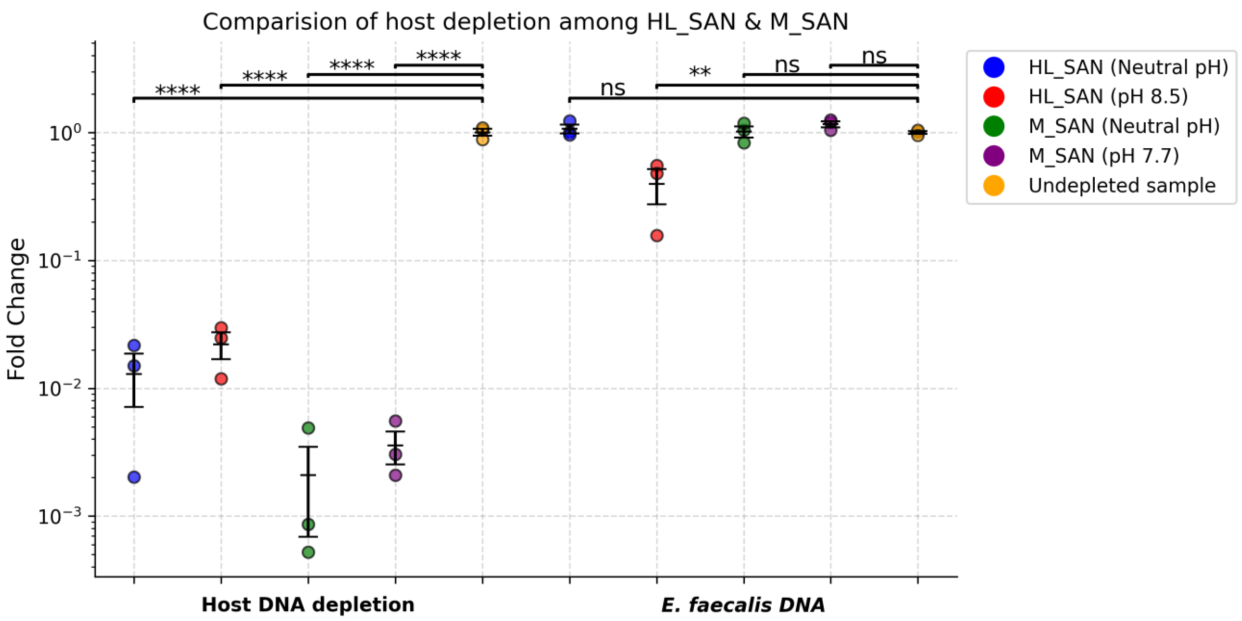

Supplementary Figure 2: Relative quantification of the host and the *Enterococcus faecalis* DNA in urine samples spiked with WBC and *E. faecalis* and subjected to depletion by HL_SAN (252 U) and M_SAN (292 U) at two different pH conditions (represented by different colors) to determine their respective depletion abilities. The values are normalized by determining the fold change calculated by the difference with the undepleted control. All the test values are mean ± SD, with n=3 biological replicates for each condition. ns > 0.05, *p ≤ 0.05, **p ≤ 0.01. ***p ≤ 0.001, ****p ≤ 0.0001. Source data are provided as a Source Data file.

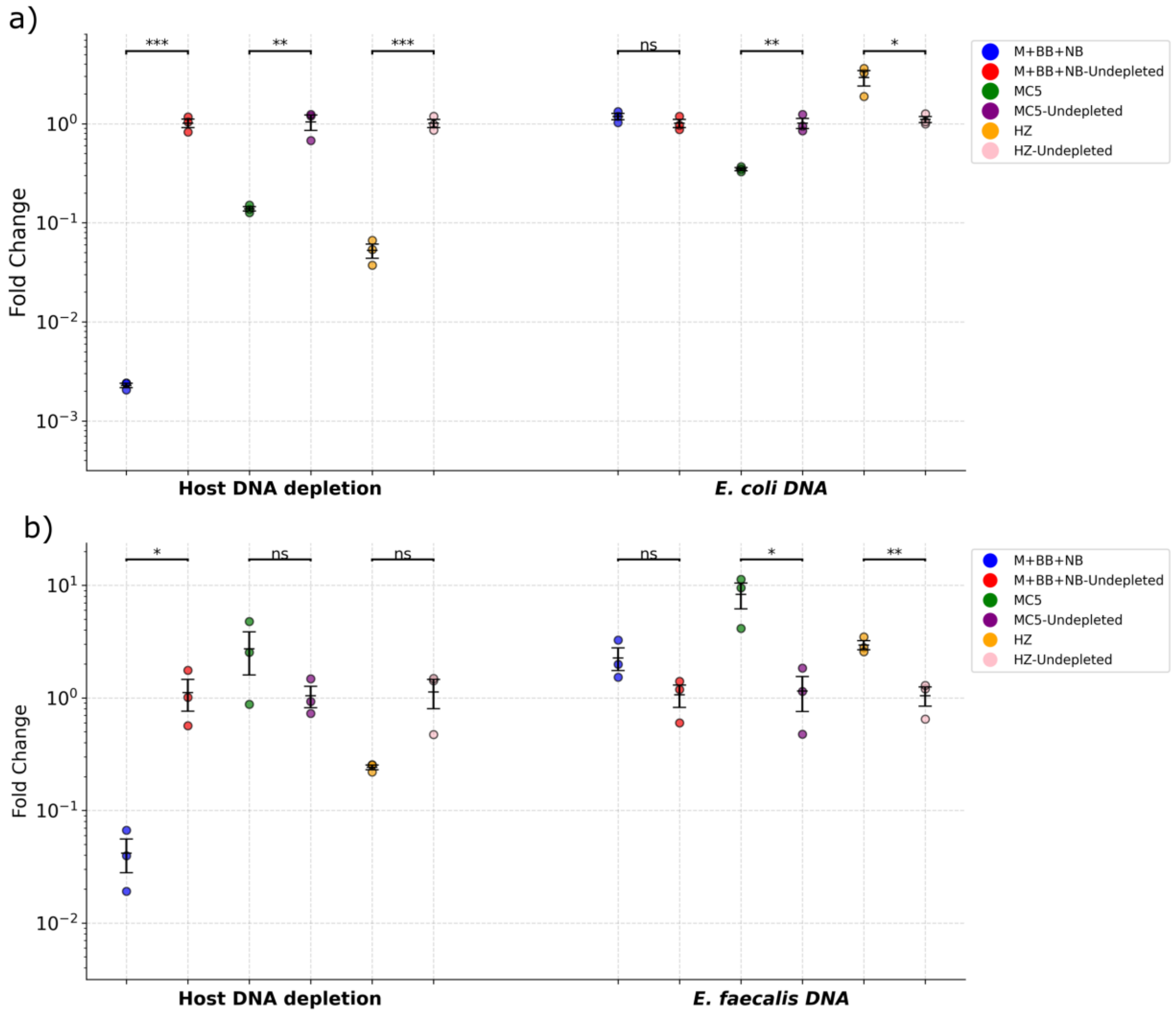

Supplementary Figure 3: Relative quantification of the host and the spiked bacterial DNA in urine samples spiked with WBC and subjected to depletion by different extraction methodologies (represented by different colors) to determine their respective depletion abilities. Two separate sample sets were spiked with *Escherichia coli* (a) and *Enterococcus faecalis* (b) at clinically relevant concentrations of 10^5 CFU/mL. The values are normalized by determining the fold change calculated by the difference with the undepleted control. All the test values are mean ± SD, with n=3 biological replicates for each condition. ns > 0.05, *p ≤ 0.05, **p ≤ 0.01. ***p ≤ 0.001, ****p ≤ 0.0001. Abbreviations: MC5: Molysis Complete 5, HZ: Host Zero Microbial DNA extraction kit, M+BB+NB: optimized in-house method. Source data are provided as a Source Data file.

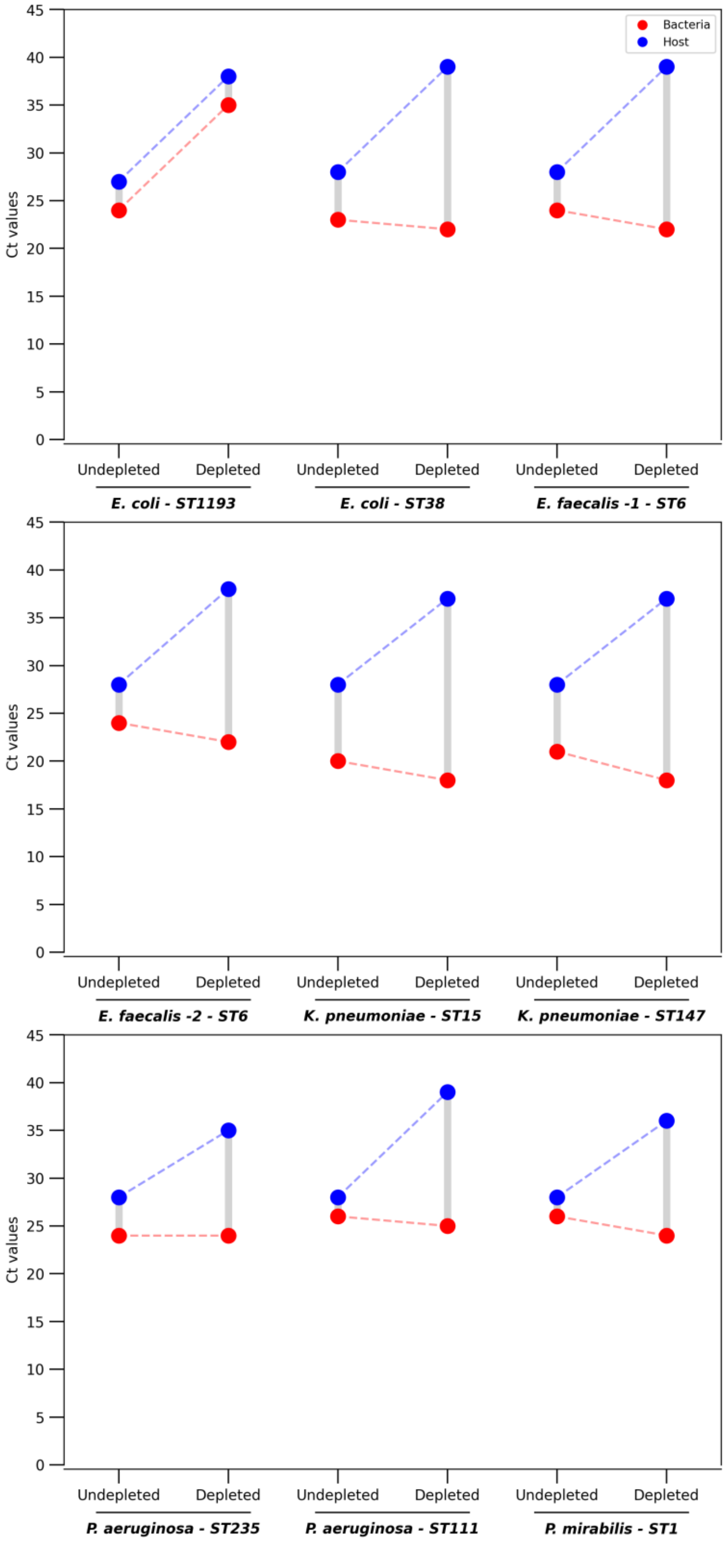

Supplementary Figure 4: host depletion performance of the optimized method across prevalent uropathogenic sequence types. Relative quantification of the host and the spiked bacterial DNA in urine samples spiked with WBC and subjected to depletion by the optimized method. A single batch of healthy urine sample was first spiked with WBC at a concentration of 5.84×10^4 cells/mL, and then 18 aliquots were made prior to spiking the different clinical sequence type strains. The bacterial (red dots)

and host DNA (blue dots) in the depleted and undepleted samples were quantified using primers targeting the *β-actin* gene and respective housekeeping genes, described in Supplementary Data 5. The Ct values of the host increased by an average of 9.74 cycles (≈10^3 fold) after the saponin treatment, while the bacterial DNA was slightly enriched by 1.6 cycles. The *Escherichia coli* ST1193 is the only exception, as the bacterial DNA loss was observed in the depleted sample. Source data are provided as a Source Data file.

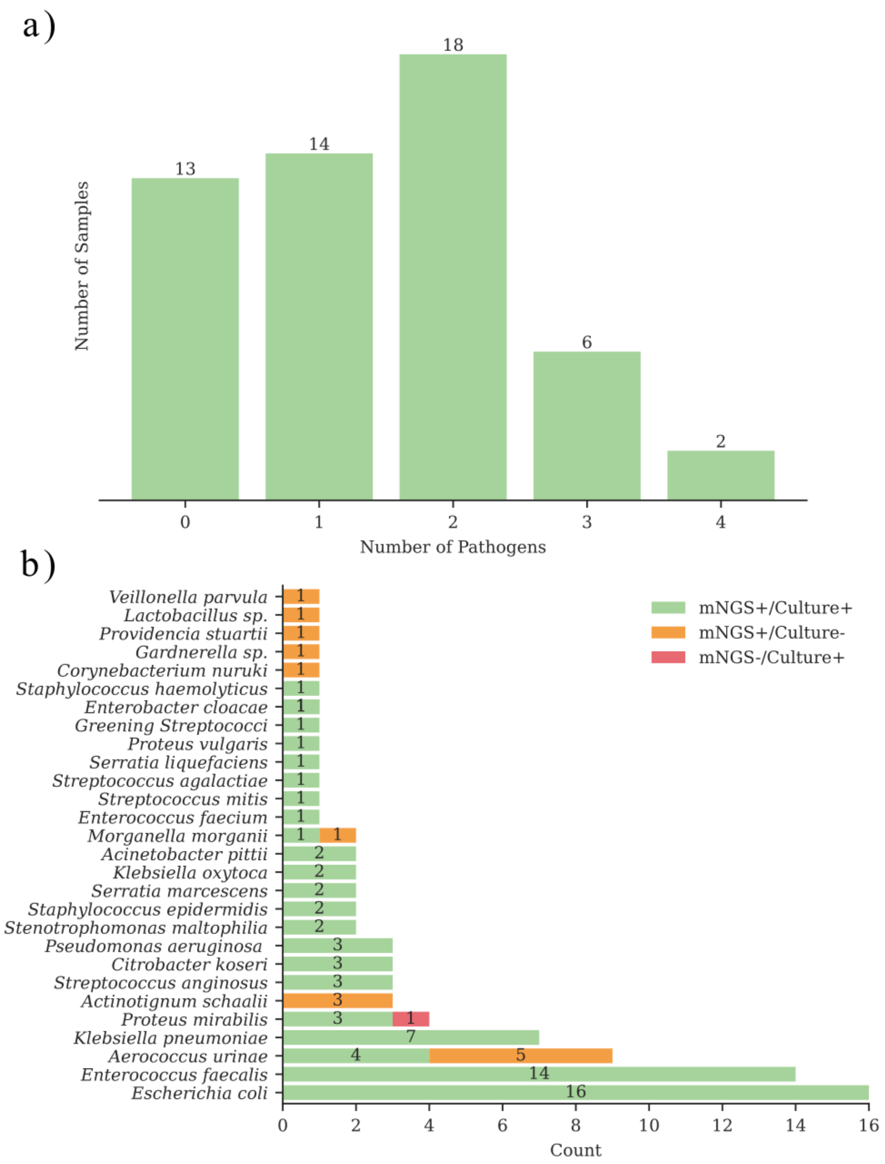

Supplementary Figure 5: Summary of the pathogen identification results for the optimized method. Subfigure (a) histogram shows the number of samples with a certain number of pathogens, as identified through routine culturing (MALDI-TOF). A value of 0 indicates culture-negative samples (13). Samples with one pathogen (14) were classified as mono-microbial, while those with more than one pathogen (26) were considered polymicrobial. Subfigure (b) illustrates the frequency of identification for each species. Green bars represent samples where the species was found through both routine culturing and mNGS, orange bars denote samples identified through mNGS and later verified using molecular methods, and red bars indicate samples where the species was identified exclusively through routine culturing. Source data are provided as a Source Data file.

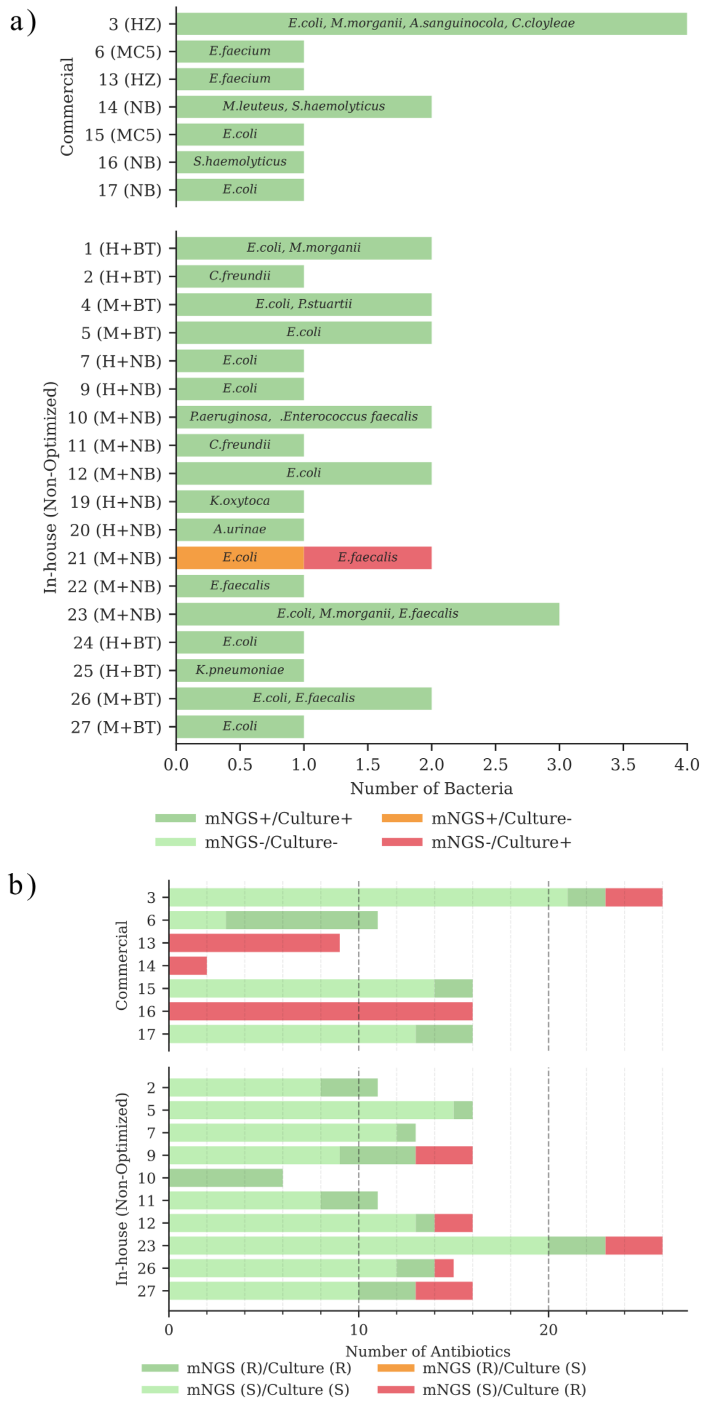

Supplementary Figure 6: Overview of pathogen identification and antibiotic susceptibility benchmarking for non-optimized in-house methods. Subfigure (a) presents benchmarking of species identification by mNGS against routine testing (MALDI-TOF). Dark green bars represent species identified through both routine testing and mNGS (concordant). Light Green bars indicate the samples found to be negative through both routine testing and mNGS (concordant). Orange bars highlight those species uniquely identified by mNGS, and red bars denote species uniquely identified by routine testing. Subfigure (b) presents concordance between mNGS results and routine AST (VITEK-2). Dark green bars indicate both routine AST and mNGS results suggest resistance, whereas light green bars indicate both routine AST and mNGS indicate susceptibility. Red indicates cases where resistance was identified in routine AST, but no corresponding resistance mechanism was detected through mNGS (false

negative). Orange bars denote detected ARGs without phenotypic resistance to the corresponding antibiotic. Source data are provided as a Source Data file.

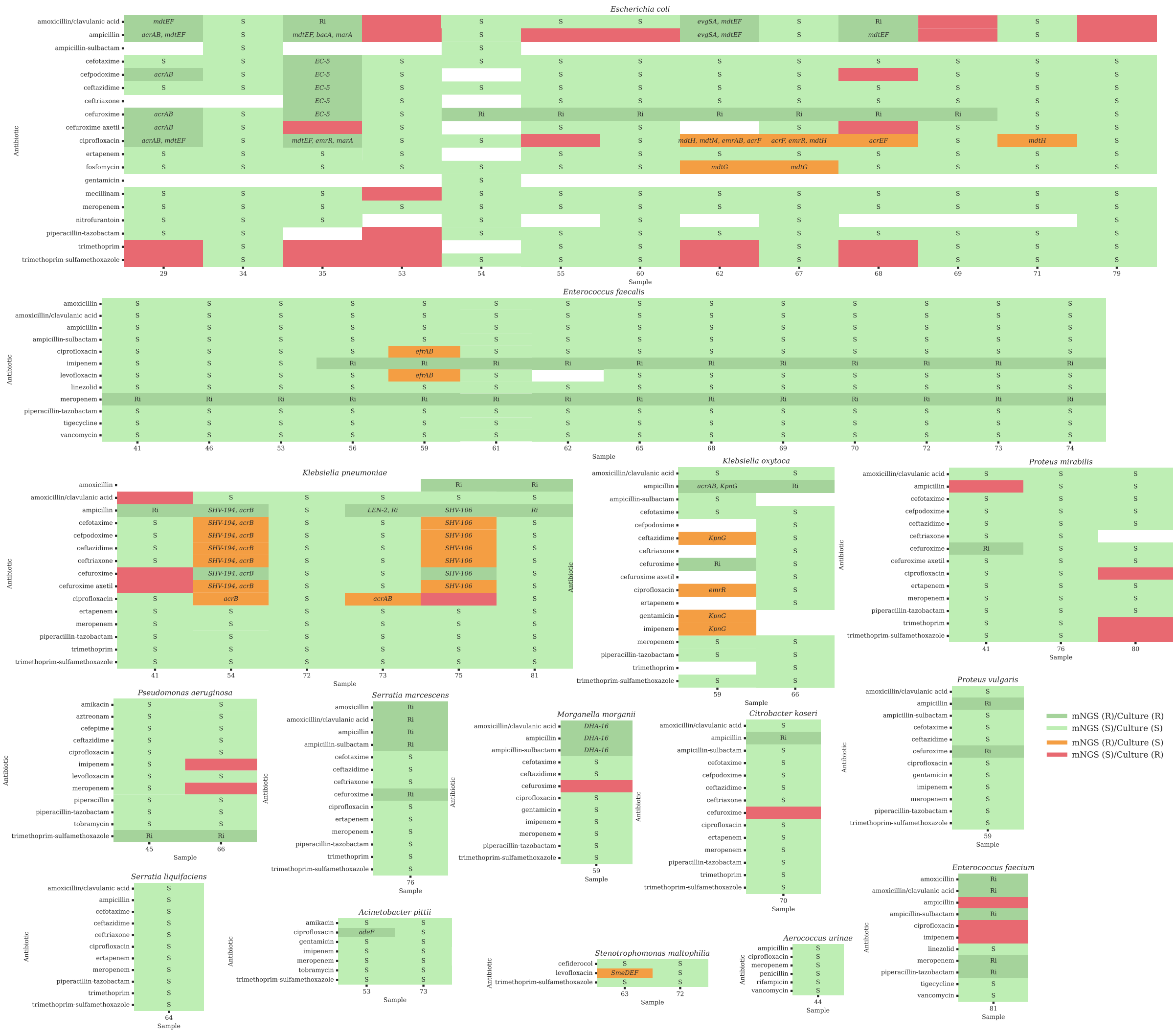

Supplementary Figure 7: Detailed overview of AST benchmarking results for the optimized method. Heatmaps show the concordance between routine antimicrobial susceptibility testing (VITEK-2) results and metagenomic (mNGS) resistance predictions derived from antimicrobial resistance gene (ARG) data for samples processed using the optimized method. The data are grouped by pathogen and displayed per antibiotic for each UTI sample. Green cells indicate concordance between observed phenotypes and detected ARGs (true positives or negatives). Light green cells indicate both routine AST and mNGS indicated sensitivity towards the antibiotic, whereas dark green cells indicate both methods indicate resistance. The cells are annotated with 'S' for susceptibility, 'Ri' for inferred or intrinsic resistance, and the ARG name when resistance mechanisms are detected. Red cells indicate instances where resistance was identified in routine AST, but no corresponding resistance mechanism was detected through mNGS (false negative). Orange cells represent detected ARGs without corresponding phenotypic resistance to the relevant antibiotic, and these cells are annotated with the ARG names. AMR predictions were made exclusively for antibiotics present in both the phenotypic and genotypic datasets. Source data are provided as a Source Data file.

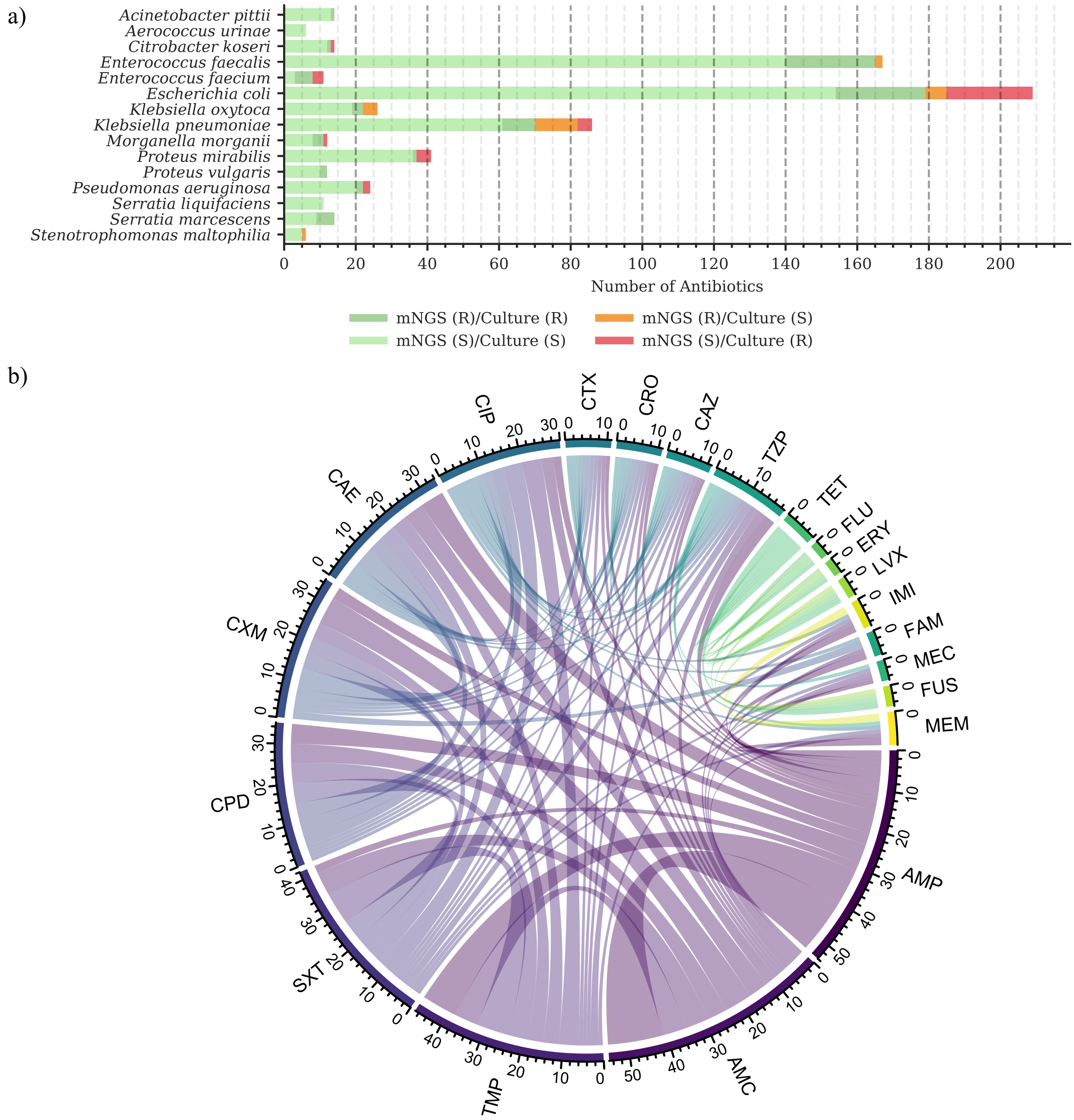

Supplementary Figure 8: AST benchmarking concordance for the optimized method stratified by species and co-occurrence of antibiotic resistance among the samples. Subfigure (a) Overview of the concordance between routine antimicrobial susceptibility testing (VITEK-2) results and metagenomic (mNGS) resistance predictions derived from antimicrobial resistance gene (ARG) data for samples processed using the optimized method. Green bars indicate concordance between observed phenotypes and detected ARGs (light green indicates true susceptibility and dark green indicates true resistance). Red bars highlight instances where resistance was identified in routine AST, but no corresponding resistance mechanism was detected through mNGS (false negatives). Orange bars signify detected ARGs without phenotypic resistance to the corresponding antibiotic. AMR predictions were made exclusively for antibiotics present in both the phenotypic and genotypic datasets. Subfigure (b) shows a chord diagram depicting the co-occurrence of antibiotic resistance in pathogens from samples processed via the optimized method. Each antibiotics sector is annotated with absolute numbers to clarify the number of pathogens in which resistance to each antibiotic occurs. The size of the connections between sectors (the ribbon's thickness) reflects how frequently resistance to the connected antibiotics appears in a common pathogen. Colors are used to visually distinguish these connections. A detailed overview of the results can be found in Supplementary Data 1. Source data are provided as a Source Data file.

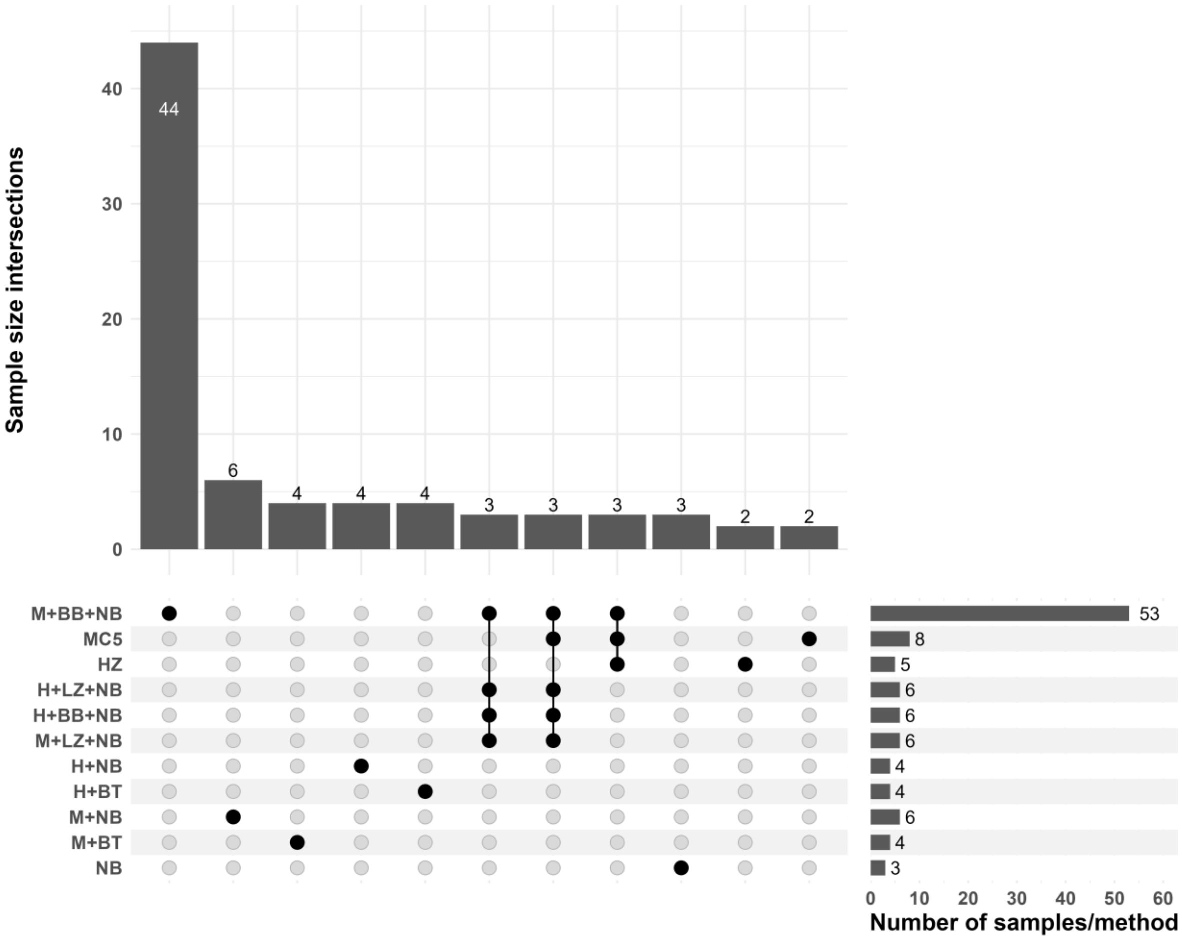

Supplementary Figure 9: An upset plot illustrating the shared and unique samples among the included methods. The upper panel shows the number of samples shared across the methods, while the dots in the lower panel indicate the unique intersections of samples. The connected dots highlight all intersection combinations. The vertical bars on the lower right represent the samples tested with each individual method. For example, of the total 53 samples tested with M+BB+NB, 44 were unique and tested solely with this method, while three samples were extracted with all M+BB+NB, MC5 & HZ, as shown by the connecting dots. Source data are provided as a Source Data file.

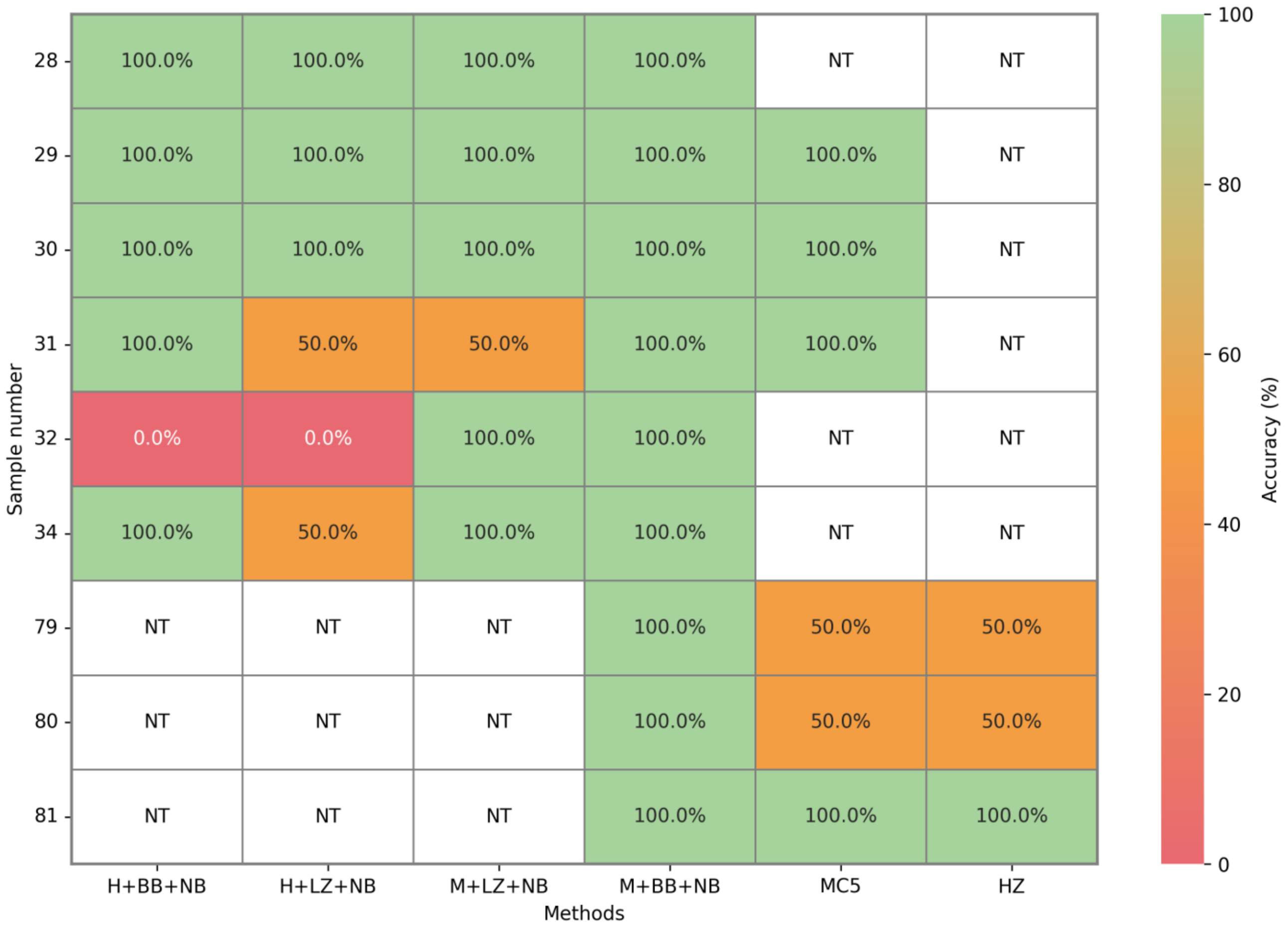

Supplementary Figure 10: Overview of pathogen identification benchmarking accuracies for direct method comparison. Heatmap indicating the accuracy of pathogen identification of the methods H+BB+NB, H+LZ+NB, M+LZ+NB, M+BB+NB, MC5, and HZ across nine samples. The pathogen identification accuracy was calculated for each of the methods and sample by benchmarking the mNGS results against those obtained from clinical routine (MALDI-TOF). Accuracy scores are colored based on the color scale (right) and the percentage values are annotated in each cell. 'NT' indicates the method was not tested on the specific sample denoted on the y-axis. Source data are provided as a Source Data file.

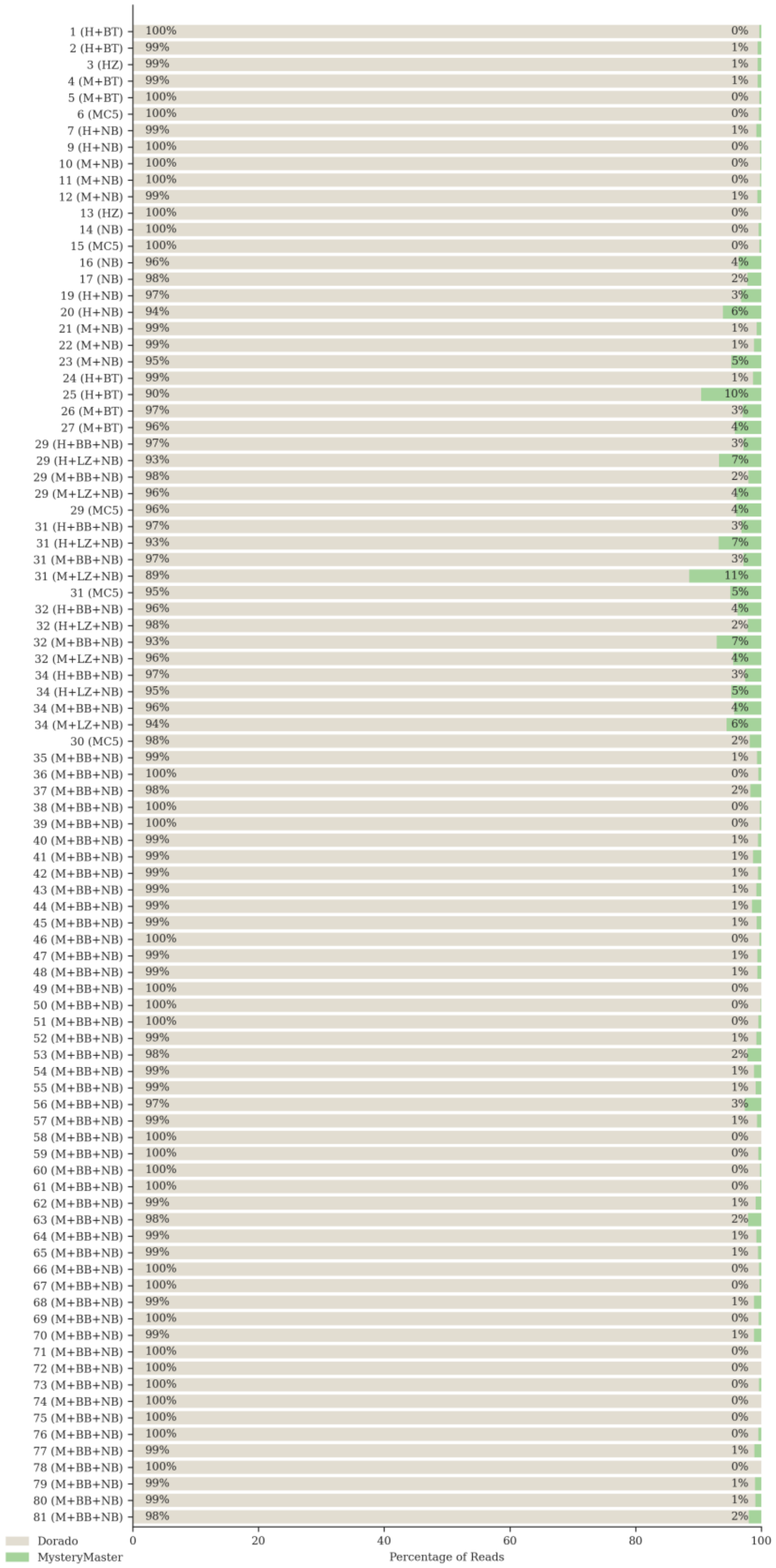

Supplementary Figure 11: Demultiplexing overview. Bar graphs illustrating the percentage of sequencing reads classified by Dorado and those unclassified, which MysteryMaster recovered. Green indicates the reads recovered by MysteryMaster. The absolute percentages are annotated where the number on the left are the percentage of reads classified by Dorado and the numbers on the right are those classified by MysteryMaster. Source data are provided as a Source Data file.

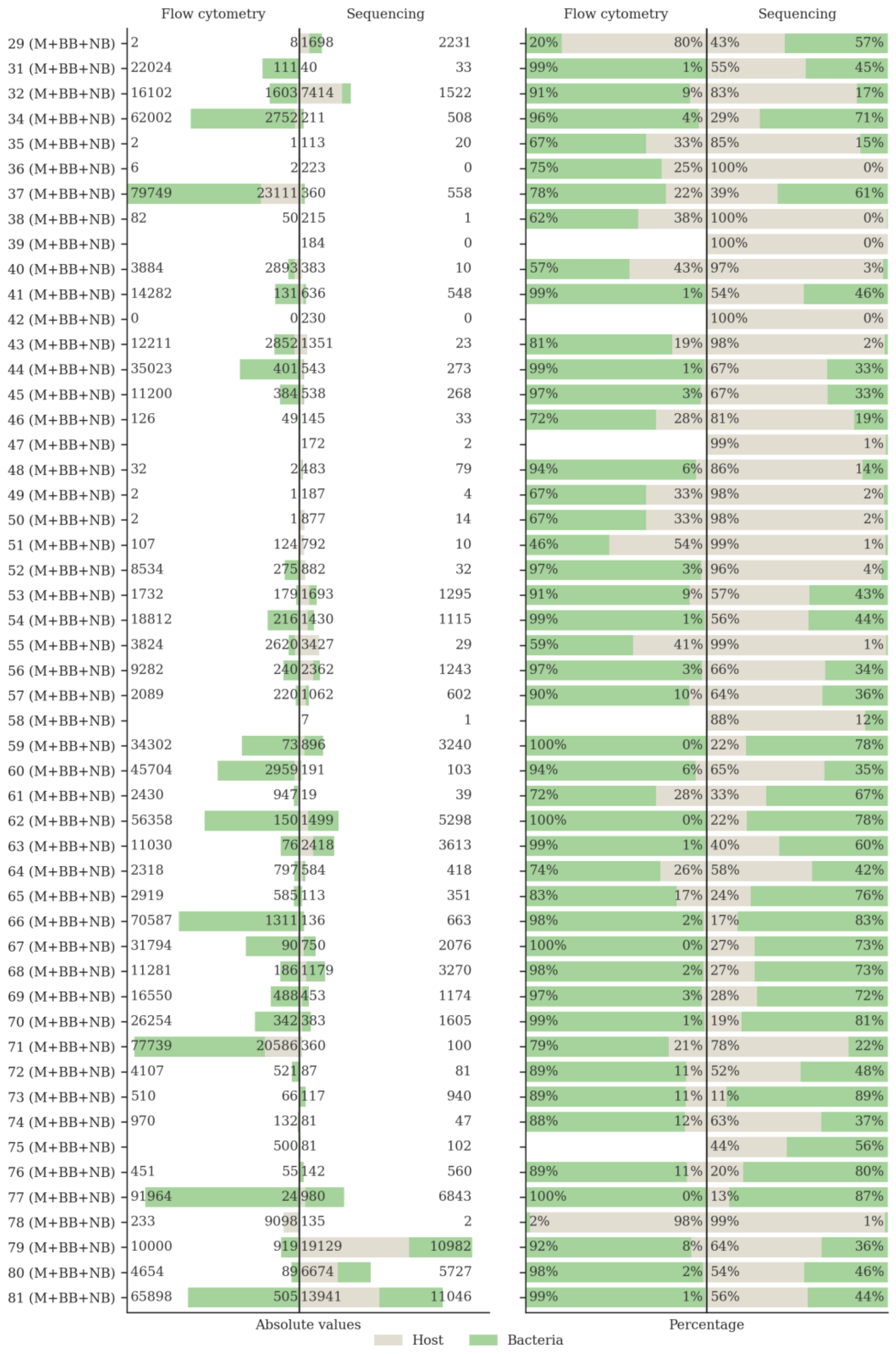

Supplementary Figure 12: Correlation between flow cytometry and mNGS abundance. The tornado charts illustrate the relationship between host (in grey) and bacterial cells (in light green) based on flow cytometry data (left), compared to host-to-bacterial reads (right) obtained through metagenomic next-generation sequencing (mNGS). The right panel displays the normalized data, while the left panel presents the absolute data. Together, these plots enhance our understanding of the effectiveness of various methodologies across a range of clinical samples for the in-house optimized method. The tornado charts are annotated with absolute numbers, where the numbers nearest the center of the tornado

represent the host data and those nearest the outside represent the bacterial data. Source data are provided as a Source Data file.

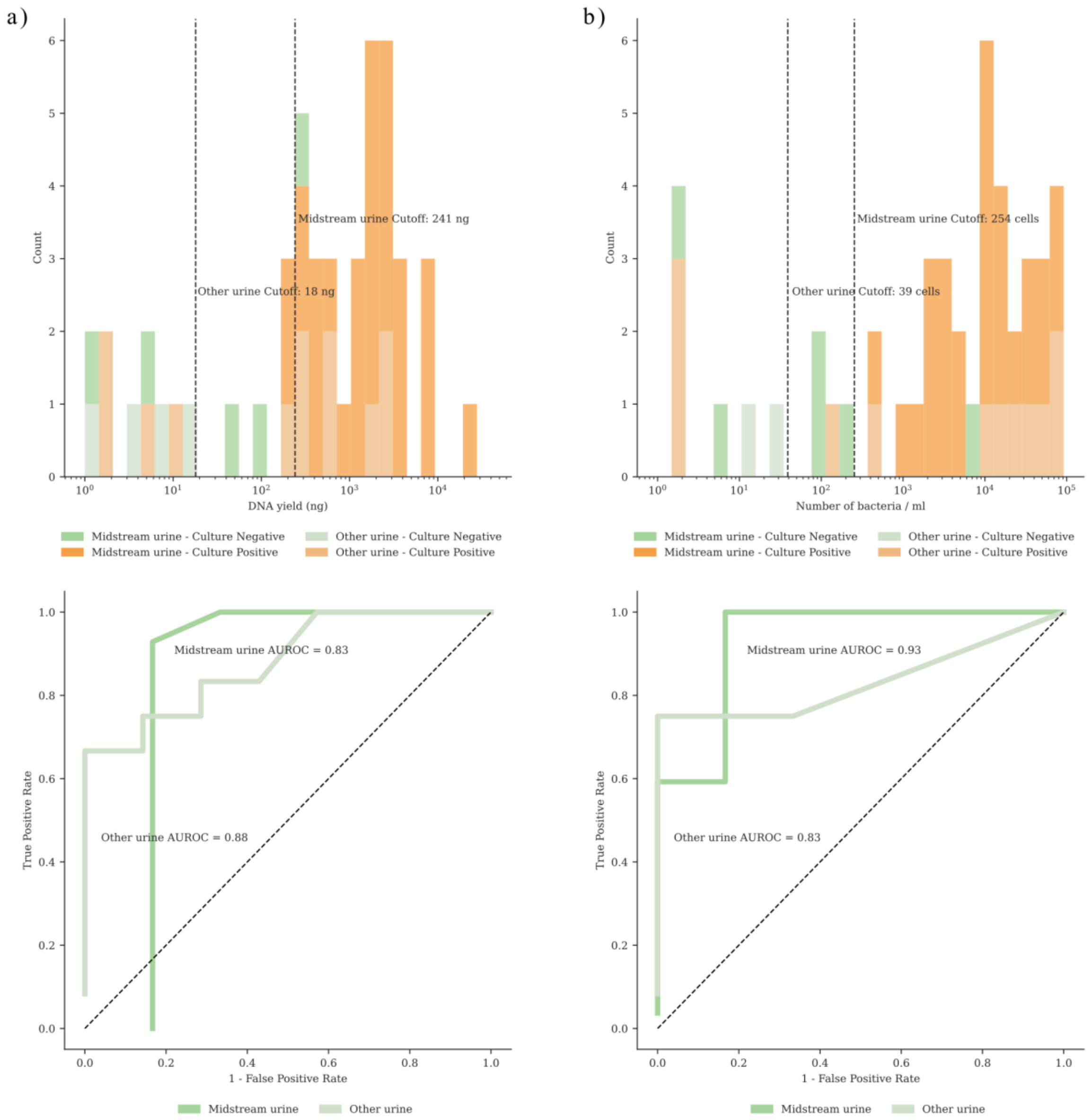

Supplementary Figure 13: Analysis of DNA yield and bacterial cell receiver operating characteristic (ROC) curve, divided by midstream urine and other urine. The analysis was performed on samples extracted using the in-house optimized method (n=53 urine samples). Subfigure (a) presents the results for DNA yield, while (b) displays the bacterial cell count per mL identified through flow cytometry. The histogram (top) illustrates the distribution of each variable for culture-positive and culture-negative samples, emphasizing the optimal cutoff for distinguishing between these samples. Colors indicate whether the samples are from midstream urine or other urine. The ROC curve (bottom) is annotated with the area under the ROC curve (AUROC) for each variable. Source data are provided as a Source Data file.

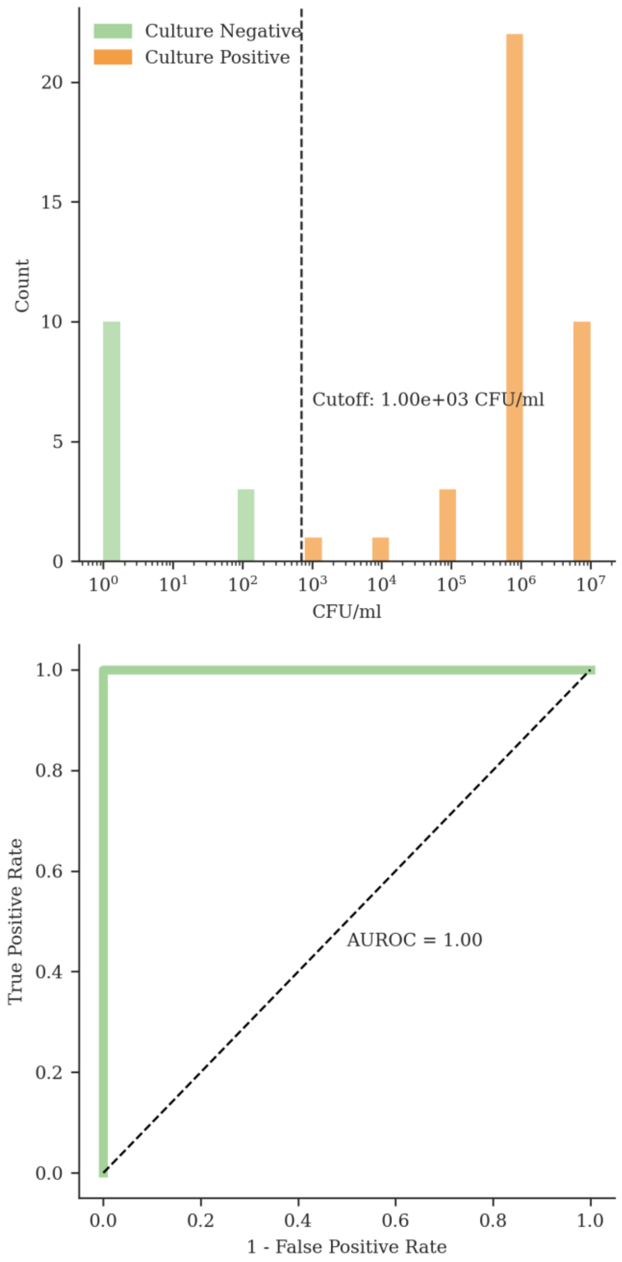

Supplementary Figure 14: Analysis of CFU/mL receiver operating characteristic (ROC) curve. The analysis was performed on samples extracted using the in-house optimized method (n=53 urine samples). The histogram (top) illustrates the distribution of culture-positive (orange) and culture-negative (green) samples by CFU/mL. The ROC curve (bottom) is annotated with the area under the ROC curve (AUROC). Source data are provided as a Source Data file.

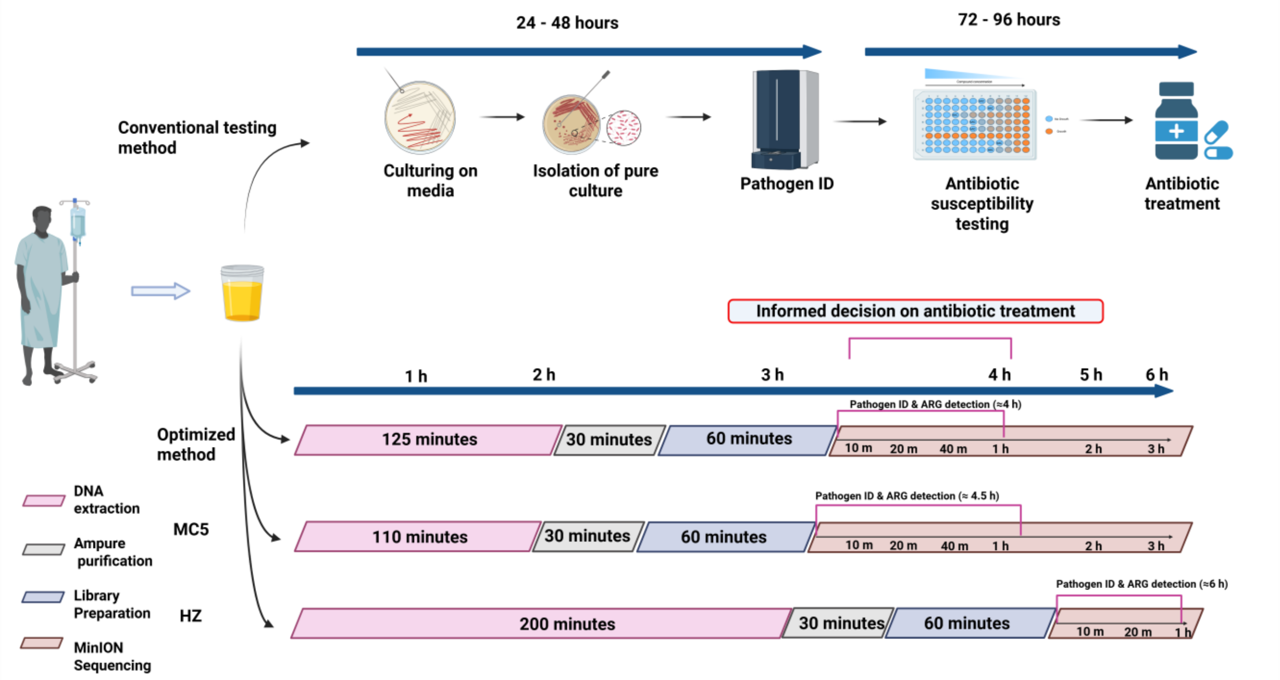

Supplementary Figure 15: Comparison of the total turnaround time between the optimized method and the commercial methods (MC5 and HZ) for the clinical samples. The timeline for the method is divided and color-coded according to DNA extraction, purification, library preparation, and sequencing. Overall, the optimized method achieved a turnaround time of approximately 4 hours for determining the antibiotic treatment. In contrast, MC5 and HZ had turnaround times of 4.5 hours and 6 hours, respectively. Created in BioRender. Branders, S. (2025) https://BioRender.com/z06y557.

References:

1. Bellankimath, A. B. *et al.* Culture and amplification-free nanopore sequencing for rapid detection of pathogens and antimicrobial resistance genes from urine. *Eur J Clin Microbiol Infect Dis* (2024) doi:10.1007/s10096-024-04929-1.
