# Supplementary material for: Metagenomic sequencing enables accurate pathogen and antimicrobial susceptibility profiling in complicated UTIs in approximately four hours
Source: Nat Commun. 2025 Dec 3;17:187. doi: 10.1038/s41467-025-66865-8 (PMC12780005; doi:10.1038/s41467-025-66865-8)
Supplement: Supplementary file 2 — Description of Additional Supplementary Files [file 41467_2025_66865_MOESM2_ESM.pdf]

Metagenomic sequencing enables accurate pathogen and antimicrobial susceptibility profiling in complicated UTIs in approximately four hours

Anurag Basavaraj Bellankimath[1#], Sverre Branders[1#], Isabell Kegel[2,3], Jawad Ali[1], Fatemeh Asadi[1], Truls E. Bjerklund Johansen[5,6], Can Imirzalioglu[2,3], Torsten Hain[2,3], Florian Wagenlehner[3,4], and Rafi Ahmad[1,7*]

[1]Department of Biotechnology, Inland Norway University of Applied Sciences, Holsetgata 22, 2317, Hamar, Norway.

[2]Institute of Medical Microbiology, Justus Liebig University Giessen, Giessen, Germany

[3]German Center for Infection Research (DZIF), Partner Site Giessen-Marburg-Langen, Giessen, Germany

[4]Clinic for Urology, Pediatric Urology and Andrology, Justus Liebig University Giessen, Giessen, Germany

[5]Institute of Clinical Medicine, University of Oslo, Norway

[6]Institute of Clinical Medicine, University of Aarhus, Denmark

[7]Institute of Clinical Medicine, Faculty of Health Sciences, UiT - The Arctic University of Norway, Hansine Hansens veg 18, 9019, Tromsø, Norway

Email: anurag.bellankimath@inn.no, sverre.branders@inn.no , isabell.kegel@med.uni-giessen.de, jawad.ali@inn.no, t.e.b.johansen@medisin.uio.no, can.imirzalioglu@mikrobio.med.uni-giessen.de , torsten.hain@mikrobio.med.uni-giessen.de , florian.wagenlehner@chiru.med.uni-giessen.de , rafi.ahmad@inn.no[*]

Supplementary Data 1: An overview of the metagenomic sequencing results. The table includes the method, sample volume used, bacteria to host cell number ratio (flow cytometry), the total number of generated reads, host reads, and number of identified reads per pathogen for the clinical samples.

Supplementary Data 2: An overview of routine microbiological results. For each sample, the patient gender, pathogens identified using MALDI-TOF, their number of colony forming units (CFU) / mL, and their resistance profile, identified using VITEK-2, is given.

Supplementary Data 3: An overview of the flow cytometry results. The number of different cells (erythrocytes, leukocytes, bacteria, round epithelia, squamous epithelia, and yeast) measured by flow cytometry are given. The total number of host cells and ratio of bacterial to host cells is also given. The culture results column indicates whether conventional microbial culturing was positive (TRUE) or negative (FALSE). Results are given for each sample, and the method used was annotated in brackets in the sample ID.

Supplementary Data 4: Calculation of per sample cost for each method. The cost calculation is broken down into cost of DNA extraction (broken up into different reagents used per method), sequencing flow cell, and library preparation reagents for different Nanopore platforms.
