# Supplementary material for: Metagenomic sequencing enables accurate pathogen and antimicrobial susceptibility profiling in complicated UTIs in approximately four hours
Source: Nat Commun. 2025 Dec 3;17:187. doi: 10.1038/s41467-025-66865-8 (PMC12780005; doi:10.1038/s41467-025-66865-8)
Supplement: Supplementary file 7 — Reporting Summary [file 41467_2025_66865_MOESM7_ESM.pdf]

nature portfolio

Corresponding author(s): Rafi Ahmad

Last updated by author(s): Nov 9, 2025

Reporting Summary

Nature Portfolio wishes to improve the reproducibility of the work that we publish. This form provides structure for consistency and transparency in reporting. For further information on Nature Portfolio policies, see our Editorial Policies and the Editorial Policy Checklist.

Statistics

For all statistical analyses, confirm that the following items are present in the figure legend, table legend, main text, or Methods section.

n/a	Confirmed	
☐	☒	The exact sample size (*n*) for each experimental group/condition, given as a discrete number and unit of measurement
☐	☒	A statement on whether measurements were taken from distinct samples or whether the same sample was measured repeatedly
☐	☒	The statistical test(s) used AND whether they are one- or two-sided<br>*Only common tests should be described solely by name; describe more complex techniques in the Methods section.*
☒	☐	A description of all covariates tested
☒	☐	A description of any assumptions or corrections, such as tests of normality and adjustment for multiple comparisons
☐	☒	A full description of the statistical parameters including central tendency (e.g. means) or other basic estimates (e.g. regression coefficient) AND variation (e.g. standard deviation) or associated estimates of uncertainty (e.g. confidence intervals)
☐	☒	For null hypothesis testing, the test statistic (e.g. *F*, *t*, *r*) with confidence intervals, effect sizes, degrees of freedom and *P* value noted<br>*Give P values as exact values whenever suitable.*
☒	☐	For Bayesian analysis, information on the choice of priors and Markov chain Monte Carlo settings
☒	☐	For hierarchical and complex designs, identification of the appropriate level for tests and full reporting of outcomes
☒	☐	Estimates of effect sizes (e.g. Cohen's *d*, Pearson's *r*), indicating how they were calculated

Our web collection on statistics for biologists contains articles on many of the points above.

Software and code

Policy information about availability of computer code

Data collection	Metagenomic sequencing data was collected using MinKNOW version 6.0.11
Data analysis	Software used in the study are all publically available or can be accessed at the Nanopore resources through the Oxford Nanopore Technologies webpage. Please refer to the methodology section for more details

For manuscripts utilizing custom algorithms or software that are central to the research but not yet described in published literature, software must be made available to editors and reviewers. We strongly encourage code deposition in a community repository (e.g. GitHub). See the Nature Portfolio guidelines for submitting code & software for further information.

Data

Policy information about availability of data

All manuscripts must include a data availability statement. This statement should provide the following information, where applicable:

- Accession codes, unique identifiers, or web links for publicly available datasets
- A description of any restrictions on data availability
- For clinical datasets or third party data, please ensure that the statement adheres to our policy

All the sequencing data is available on the European Nucleotide Archive (ENA) under study accession number PRJEB83412. The study data has been released publicly and can be accessed through the accession number.

Research involving human participants, their data, or biological material

Policy information about studies with human participants or human data. See also policy information about sex, gender (identity/presentation), and sexual orientation and race, ethnicity and racism.

Reporting on sex and gender	*Use the terms sex (biological attribute) and gender (shaped by social and cultural circumstances) carefully in order to avoid confusing both terms. Indicate if findings apply to only one sex or gender; describe whether sex and gender were considered in study design; whether sex and/or gender was determined based on self-reporting or assigned and methods used. Provide in the source data disaggregated sex and gender data, where this information has been collected, and if consent has been obtained for sharing of individual-level data; provide overall numbers in this Reporting Summary. Please state if this information has not been collected. Report sex- and gender-based analyses where performed, justify reasons for lack of sex- and gender-based analysis.*
Reporting on race, ethnicity, or other socially relevant groupings	*Please specify the socially constructed or socially relevant categorization variable(s) used in your manuscript and explain why they were used. Please note that such variables should not be used as proxies for other socially constructed/relevant variables (for example, race or ethnicity should not be used as a proxy for socioeconomic status). Provide clear definitions of the relevant terms used, how they were provided (by the participants/respondents, the researchers, or third parties), and the method(s) used to classify people into the different categories (e.g. self-report, census or administrative data, social media data, etc.) Please provide details about how you controlled for confounding variables in your analyses.*
Population characteristics	The sample cohort consisted of 78 samples of which approximately 38% the samples were from female and 62% from male patients.
Recruitment	The left over urine sample from the suspected UTI patients was used. The only screening criteria for the selection of samples was the presence of leukocytes that potentially indicates a urinary tract infection.
Ethics oversight	The study received ethical approval from the Ethics Committee of Justus Liebig University Giessen, Faculty of Medicine (AZ 158/20) for patients with UTIs and from the regional committees for medical and health research ethics (REC Helse Sør-Øst) (691177) for the healthy donors.

Note that full information on the approval of the study protocol must also be provided in the manuscript.

Field-specific reporting

Please select the one below that is the best fit for your research. If you are not sure, read the appropriate sections before making your selection.

☒ Life sciences ☐ Behavioural & social sciences ☐ Ecological, evolutionary & environmental sciences

For a reference copy of the document with all sections, see nature.com/documents/nr-reporting-summary-flat.pdf

Life sciences study design

All studies must disclose on these points even when the disclosure is negative.

Sample size	The current study is a proof-of-concept investigation evaluating the performance of 11 different techniques for culture-independent DNA extraction directly from clinical urine samples. A total of 78 samples were tested across the 11 methods, with 53 tested exclusively using the optimized in-house method. A more extensive validation study is currently underway in which the optimized in-house method is being tested on over 250 urine samples. No statistical test were used to determine sample size.
Data exclusions	No data was excluded from the study.
Replication	The qPCR experiments to determine host depletion among the methods were conducted in triplicate (see the paper for design and results). All the replication extractions were successful. All extraction and sequencing experiments on the spike-in samples were performed once. Similarly, all the clinical samples were extracted and sequenced once and then compared with the conventional microbiological results for correlation.
Randomization	Excess urine samples were collected from patients suspected of having a urinary tract infection (UTI) in the urology ward for the study. All samples gathered during the study were collected randomly on different days and at various times. The only criterion for sample selection was the presence of leukocytes in the dipstick or flow cytometry data, as this indicates a potential urinary tract infection. No leukocyte count cutoff was applied to further screen the samples.
Blinding	The routine microbiological data, i.e., microbial culture results and antibiotic susceptibility results, were not disclosed until the metagenomic results were analyzed. The sample allocation to an extraction method group was at random without any prior information on the pathogen number or the type.

Reporting for specific materials, systems and methods

We require information from authors about some types of materials, experimental systems and methods used in many studies. Here, indicate whether each material, system or method listed is relevant to your study. If you are not sure if a list item applies to your research, read the appropriate section before selecting a response.

Materials & experimental systems

n/a	Involved in the study
☒	☐ Antibodies
☒	☐ Eukaryotic cell lines
☒	☐ Palaeontology and archaeology
☒	☐ Animals and other organisms
☒	☐ Clinical data
☒	☐ Dual use research of concern
☒	☐ Plants

Methods

n/a	Involved in the study
☒	☐ ChIP-seq
☒	☐ Flow cytometry
☒	☐ MRI-based neuroimaging

Plants

Seed stocks	*Report on the source of all seed stocks or other plant material used. If applicable, state the seed stock centre and catalogue number. If plant specimens were collected from the field, describe the collection location, date and sampling procedures.*
Novel plant genotypes	*Describe the methods by which all novel plant genotypes were produced. This includes those generated by transgenic approaches, gene editing, chemical/radiation-based mutagenesis and hybridization. For transgenic lines, describe the transformation method, the number of independent lines analyzed and the generation upon which experiments were performed. For gene-edited lines, describe the editor used, the endogenous sequence targeted for editing, the targeting guide RNA sequence (if applicable) and how the editor was applied.*
Authentication	*Describe any authentication procedures for each seed stock used or novel genotype generated. Describe any experiments used to assess the effect of a mutation and, where applicable, how potential secondary effects (e.g. second site T-DNA insertions, mosiacism, off-target gene editing) were examined.*
